# RIPK1 prevents aberrant ZBP1-initiated necroptosis

**DOI:** 10.18632/oncotarget.13926

**Published:** 2016-12-12

**Authors:** Tom Vanden Berghe, William J. Kaiser

**Affiliations:** Departments of Microbiology, Immunology and Molecular Genetics, University of Texas Health Sciences Center, San Antonio, TX, USA; Department of Biomedical Molecular Biology, Inflammation Research Centre, VIB, Ghent University, Ghent, Belgium

**Keywords:** necroptosis, RIPK1, ZBP1, RIPK3, MLKL Autophagy

Receptor interacting protein kinase 1 (RIPK1) regulates inflammation and cell death, in host defense and homeostasis. The adaptor function of RIPK1 allows pro-survival and inflammatory signaling, while its kinase activity regulates the induction of necroptosis and apoptosis. A new level of regulation through its RIP homotypic interaction motif (RHI111) was recently discovered to suppress necroptosis and inflammation driven by the putative nucleic acid sensor protein Z-DNA binding protein 1 (ZBP1), also known as DAI.

**Figure 1:**
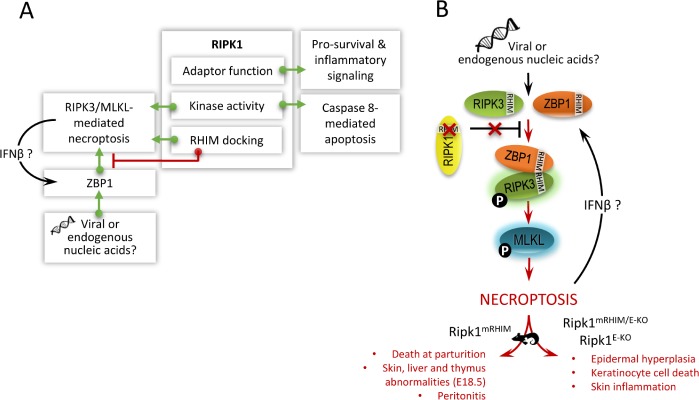
RIPK1 a master regulator of cell death and inflammation **A.** Schematic representation of the versatile functions of RIPK1. Basically, RIPK1 regulates pro-survival and inflammatory signaling in addition to apoptotic and necroptotic cell death through its different regulatory domains *viz.* adaptor, kinase and RHIM docking fnnction. **B.** Schematic representation of how the RHIM domain of RIPK1 acts as an essential break on constitutive ZBP1 activation. RIPK1 with an inactivating mutation in the RHIM that abolishes the capacity of RIPK1 to interact with other cellular RHIM containing proteins induces ZBP11RIPK3/MLKL- mediated necroptosis and inflammation. This defect signaling results in a spontaneous phenotype in the mentioned RIPK1 transgenic mice, which are briefly described. IFN, interferon; RIPK, receptor interacting protein kinase; RIPK1 mRHIM, mice expressing RIPK1 with an inactivating mutation in the RHIM; RIPK1 mRHIMtE-Ko, mice with skin specific expression of RIPK1 with an inactivating mutation in the RHIM; RIPK1 E-Ko, mice with skin specific deletion ofRIPK1; RHIM, RIP homotypic interaction motif; ZBP1, Z-DNA binding protein 1.

In the two decades following the discovery of RIPK1, RIPK1 has emerged as a master regulator of inflammatory signaling and cell death [1]. To date, important advances in our understanding of RIPK1 function continue to be uncovered. RIPK1-deficient mice fail to thrive, die within 1–3 days following birth and display extensive apoptosis. These observations prompted initial research efforts to focus on the role of RIPK1 in controlling pro-survival and inflammatory gene expression. More recently, RIPK1 kinase activity emerged as essential for coordinating death receptor-induced necrosis (Figure 1A). This type of necrosis was dubbed necroptosis upon the discovery of a chemical kinase inhibitor ofRIPK1, necrostatin-1 (Nec1). However, the model of RIPK1 kinase functioning exclusively for necroptosis proved too simplistic as kinase activity was also found to be crucial for caspase 8-mediated apoptosis under conditions where cellular inhibitors of apoptosis (ciAPs) are depleted (Figure 1A). Thus, depending on the cellular content, RIPK1 kinase can initiate either necroptosis or apoptosis. This implies that a protective phenotype observed in response to Nec1 or in the context ofRIPK1 kinase dead knockin mice (Ripk1KD mice) stems from the role of RIPK1 in regulating cell death but does not differentiate the cell death modality. Conditional deletion ofRIPK1 in mice revealed additional complexity on the regulatory action of RIPK 1. While absence of RIPK1 unleashes apoptosis in the intestinal epithelial cells [2, 3], necroptotic cell death dominates in the skin upon RIPK1 depletion [3]. RIPK1 kinase dead knock-in mice did not show any spontaneous phenotype indicating the sensitization to apoptosis in the gut upon RIPK1 deletion or necroptosis in the skin was not due to the absence of kinase activity [4, 5]. In the intestine, the absence the death domain in RIPK1 led to excessive apoptosis pointing to the essential scaffold function of this domain in RIPK1 for induction of pro-survival and inflammatory signaling (Figure 1A). However, the role of RIPK1 in preventing necroptosis in the skin remained unresolved until now.

In the current issue of Nature, both the Pasparakis and Dixit Labs independently reveal that the absence of RIPK1 in the skin promotes a necrotic phenotype driven by ZBP1, a RHIM-containing protein that has previously been shown to mediate virus-induced necroptosis through activation of RIPK311v1LKL [6, 7]. As a proof of concept, both groups generated mice expressing RIPK1 with an inactivating mutation (referred to as RIPK1mRHIM mice) in the RHIM that abolishes the capacity of RIPK1 to interact with other cellular RHIM containing proteins. Strikingly, RIPK1 mRHIM mice died around birth with enhanced cell death in the skin that correlated with robust levels of RIPK3 autophosphorylation. Blocking necroptosis with catalytically inactive RIPK3 D161N, RHIM mutant RIPK3, RIPK3 deficiency, or MLKL deficiency prevented lethality in RIPK1 mRHIMmice. Importantly, the loss of ZBP1 prevented perinatal lethality in RIPK1 mRHIM mice, unveiling an unexpected role for RIPK 1 in preventing ZBP1 dysregulation. In line with these findings, ZBP1 deficiency also prevented the development of skin lesions in mice with skin-specific depletion ofRIPK1 (Ripk1E-Ko mice) or skin-specific expression of RIPK1 mRHIM (RIPK1 mRHIMJE-Ko mice) [7]. Mechanistically, ZBP1 interacted strongly with phosphorylated RIPK3 in cells expressing RIPK1mRHIM, suggesting that the RIPK1 RHIM may prevent ZBP1 from binding and activating RIPK3 (Figure 2B). However, both studies were unable to detect constitutive interaction between RIPK1 and ZBP1. Similar to RIPK3 knock out mice [1], elimination of ZBP1, but not the other cellular RHIM-containing protein TRIF, also rescued the perinatal lethality of RIPK 1/Caspase 8-deficient mice [6]. In summary, at least three independent transgenic approaches underscore the suppressive role of RIPK1 on ZBP1- induced necroptosis and inflammation.

What could be the reason of the death at birth of Ripk1mRHIM mice? Given that Ripk1mRHIMIE-Ko mice are Viable, the lethality of RIPK1 mRHIM mice is unlikely driven by increased cell death and/or inflammation in the skin. It is tempting to speculate that in Ripk1mRHIM mice, exposure to microbes after birth initiates lethal ZBP1 / RIPK3!1v1LKL-driven inflammation similar to systemic inflammatory response syndrome (SIRS). It is remarkable that, except for the skin, no significant increase in cell death was observed in RIPK1 mRHIM mice. Undetectable levels of necroptotic cell death in cells other than the epidermis could be sufficient to initiate inflammation and consequent SIRS-induced shock. Additional research will need to distinguish whether ZBP1 functions as merely an interferon inducible RHIM-adapter protein or as a *bona fide* nucleic acid sensor surveilling RNA or DNA derived from microbes or even endogenous nucleic acids, perhaps from damaged tissue. Furthermore, additional studies will be necessary to resolve whether RIPK 1 functions as an essential brake on constitutive ZBP1 activation or as a potential sink for RIPK3 limiting activation by RIPK1. Nonetheless, these two studies have illuminated an unexpected role for ZBP1 in inflammation and the importance of RHIM interactions in both driving as well as limiting cell death.
